# Venous congestion and the geometry of Guyton

**DOI:** 10.1186/s13613-025-01593-2

**Published:** 2025-10-21

**Authors:** Jon-Emile S. Kenny, Per Werner Moller

**Affiliations:** 1 Health Sciences North Research Institute, Sudbury, ON Canada; 2 Flosonics Medical, Toronto, ON Canada; 3 Department of Anesthesia, SV Hospital Group, Institute of Clinical Sciences, Sahlgrenska Academy, University of Gothenburg, Gothenburg, Sweden

## Background

**Fig. 1:**
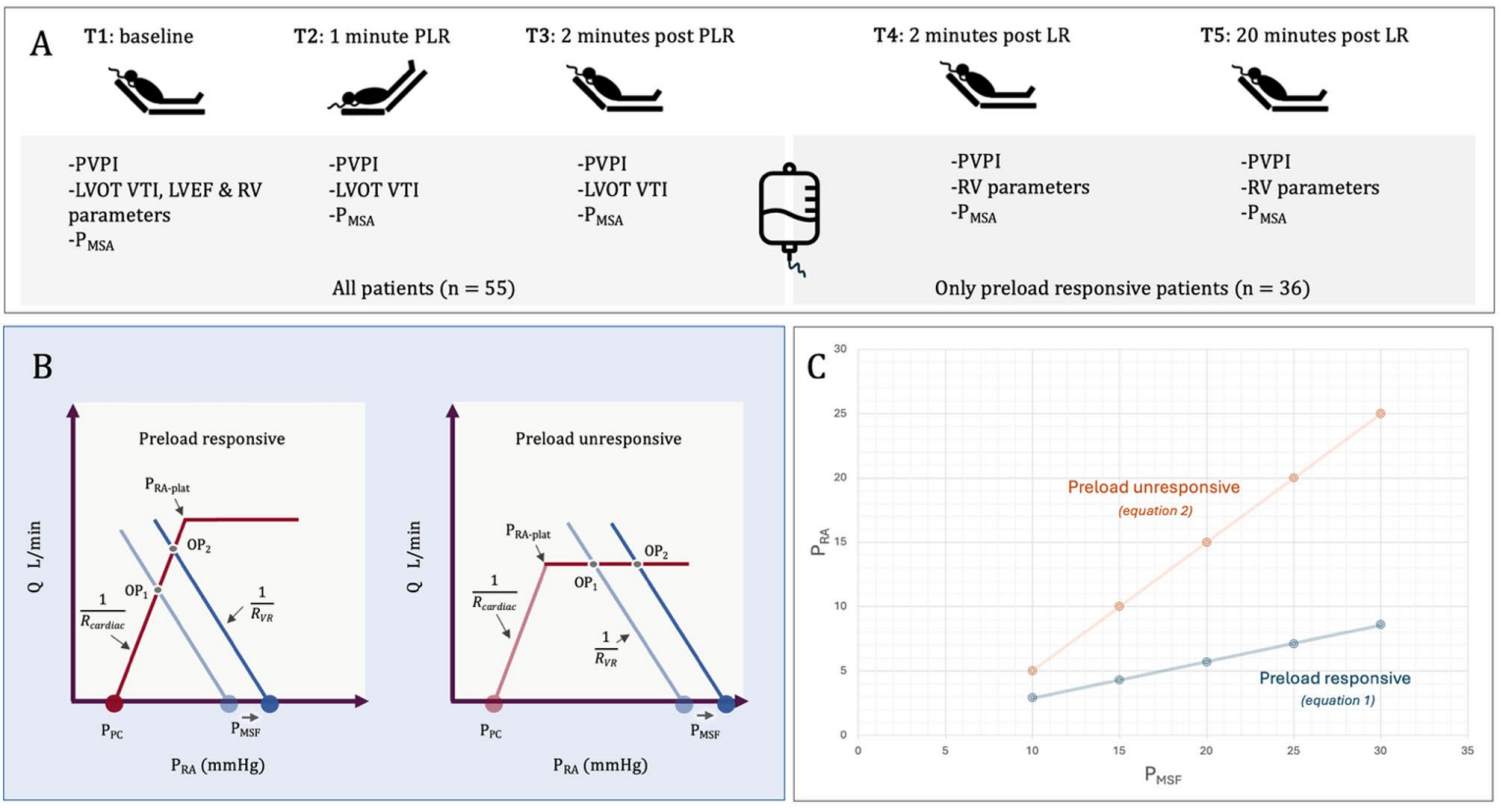
**A** Experimental protocol. Preload responsive patients were defined by a ≥ 12% increase in left ventricular outflow tract velocity time integral (LVOT VTI) during passive leg raise (PLR). Right ventricular (RV) parameters studied were 1. RV to left ventricular (LV) end-diastolic area ratio (RVEDA/LVEDA), 2. tricuspid lateral annular systolic velocity (RV S’), 3. RV fractional area change (RVFAC), 4. pulmonary acceleration time (PAT) and 5. right myocardial performance index (RIMP). **B** The Geometrical model showing effect of change in P_MSF_ on operating point (OP_1_ to OP_2_) between a preload responsive (left) and unresponsive (right) patient. P_RA−plat_ is the right atrial pressure at which the cardiac function curve plateaus **C** Using Eqs. 1 and 2, this is a graphical simulation showing effect of increasing P_MSF_ on P_RA_ (i.e., PVPI) between preload responsive and unresponsive patients assuming an R_VR_:R_CARDIAC_ ratio of 2.5 and P_RA−plat_ of 2 mmHg. PVPI portal vein pulsatility index; LVEF left ventricular ejection fraction; P_MSA_ mean systemic pressure analogue; P_MSF_ mean systemic filling pressure; Q is blood flow in liters per minute; mmHg millimeters of mercury; LR Lactated Ringer’s solution

Balan and colleagues have published an intriguing, post-hoc investigation of patients following elective coronary artery bypass grafting in *Annals of Intensive Care* [1]. They studied the interaction between a Doppler ultrasound marker of venous congestion (portal vein pulsatility index, PVPI), echocardiographic measures of right ventricular (RV) function and a surrogate of volume state, the mean systemic pressure analogue (P_MSA_). Included patients had no venous congestion (i.e., PVPI < 50%) at baseline. The experimental protocol is shown in Fig. 1A and 65% were preload responsive (PR) while 35% were preload unresponsive (PUR).

At baseline (T1), multivariable regression found that three measures of RV function (RVEDA/LVEDA, RV S’ and RIMP) associated with PVPI while P_MSA_ did not. However, for all patients across T1-T3 there was a significant correlation between P_MSA_ and PVPI. Lastly, in the subset of PR patients studied at T1, T4 and T5, P_MSA_ associated with PVPI as did the three significant RV parameters from T1.

We think that a recently proposed ‘Geometrical model’ of the circulation elaborates the findings of Balan and colleagues [2, 3]. We argue that (1) there are subtle mathematical differences in the P_MSA_-to-PVPI relation in preload responsive versus unresponsive patients and that (2) the ratio between the resistance to venous return (R_VR_) and cardiac function (i.e., cardiac resistance, R_CARDIAC_) as a measure of ‘veno-cardiac coupling’ modulates the relation between PVPI and P_MSA_.

### Synopsis of geometrical model

The Geometrical model reaffirms Guyton’s own words [4] that: “… *right atrial pressure is not one of the primary determinants of cardiac output but*,* instead*,* is itself determined simultaneously along with cardiac output*.” What, then, are the independent variables that determine right atrial pressure (P_RA_) and blood flow (Q)? The Geometrical model proposes that there are four: 1. The mean systemic filling pressure (P_MSF_), 2.) the resistance to venous return (R_VR_), 3.) the pressure surrounding the right atrium (i.e., the pericardial pressure, P_PC_), and 4.) cardiac resistance (R_CARDIAC_). Furthermore, the model specifies that the independent variables alter the P_RA_ and Q by different magnitudes contingent upon whether the patient is PR or PUR (Fig. 1B).

Because the Geometrical model asserts that both Q and P_RA_ are concurrently determined by the system of independent variables above, the model reconciles debate between the experimental approaches of Guyton and Levy [5]. Guyton altered the height of a collapsible tube, effectively using gravity to change the transmural pressure of the right atrium, analogous to changing the P_PC_; then he varied pump function (R_CARDIAC_) to return the tube wall to a trans-mural pressure of approximately zero, in which state the height of the collapsed tube was equivalent to the P_RA_. With this, Guyton iteratively generated a series of unique P_RA_-Q relationships plotted as a venous return curve. Levy, on the other hand, changed only R_CARDIAC_ to produce P_RA_-Q relationships. Regardless, neither P_RA_ nor Q in either experiment were independent variables.

### Geometrical model and venous congestion

The Geometrical model can be solved for P_RA_. To better understand the findings of Balan et al., and to simplify the analysis, we set P_PC_ to atmospheric pressure such that P_RA_ is the right atrial transmural pressure as a surrogate for PVPI. This is reasonable because increasing PVPI is partly due to greater retrograde a-waves at end-diastole, reflecting right atrial congestion into the hepatic circulation. Different equations apply for the PR and PUR states (1 and 2 respectively; see Appendix).

1 $$\:{PVPI}_{PR}\approx\:{P}_{RA}\approx\:\frac{{P}_{MSF}}{\frac{{R}_{VR}}{{R}_{CARDIAC}}+1}$$

2 $$\:{PVPI}_{PUR}\approx\:{P}_{RA}\approx\:{P}_{MSF}-\:\frac{{R}_{VR}}{{R}_{CARDIAC}}{P}_{{RA}_{plat}}$$

First, there is a direct relationship between P_MSF_ (or P_MSA_) and PVPI, but also an indirect relationship between PVPI and the ratio of the R_VR_ to R_CARDIAC_. Decreasing R_VR_ and/or increasing the R_CARDIAC_ raises P_RA_ (and therefore PVPI) for any given P_MSF_ (and vice versa). The R_VR_:R_CARDIAC_ ratio is analogous to ventriculoarterial coupling (i.e., the E_A_:E_ES_ ratio) and could partly explain the lack of association between PVPI and P_MSA_ at baseline. Consequently, the PVPI-P_MSA_ relationship must be understood in terms of ‘veno-cardiac coupling’.

Second, in PR patients, the R_VR_:R_CARDIAC_ ratio is the multiplicative inverse of PVPI while in PUR patients the ratio is the arithmetic inverse. Thus, when P_MSF_ is increased (e.g., during PLR or crystalloid infusion) the PVPI (and therefore P_RA_) is predicted to change *more* in PUR patients (Fig. 1C); conceptually this is because the venous return curve shifts right along a flat portion of the cardiac function curve (Fig. 1B).

## Limitations and conclusions

The transmural P_RA_, by itself, does not fully explain PVPI. Retrograde a-waves reflected into the hepatic and portal venous systems due to right atrial congestion is one determinant. Additionally, vascular compliance, valve function, damping properties of the hepatic venous outflow, organ compliance, perihepatic pressure, autonomic tone and reflexes such as the hepatic buffer arterial response also mediate this dynamic, waveform-based index.

We congratulate Balan and colleagues for their excellent work linking venous congestion, preload responsiveness and Guytonian principles. Though hypothesis-generating, the simplified Geometrical model connecting venous return (P_MSF_, R_VR_) and cardiac function (P_PC_, R_CARDIAC_) helps predict P_RA_ and, theoretically, venous Doppler patterns. Increased R_CARDIAC_ (i.e., impaired cardiac function) augments P_RA_ at any volume state (i.e., P_MSF_); this relationship is amplified when the patient is preload unresponsive. While not perfectly related, rising P_RA_ contributes to venous congestion and PVPI. We believe that the ability to distinguish dependent (e.g., P_RA_) from independent (e.g., R_CARDIAC_) hemodynamic variables is relevant for proper bedside therapeutic decision-making.

## Data Availability

Not applicable.

## Appendix

These equations assume two adjacent right triangles [2]: the y-axis is blood flow (Q), the x-axis is pressure (P), and the slope relating flow over pressure is 1/Resistance.

Preload responsive state:

$$\:\frac{Q}{{P}_{RA}}=\frac{1}{{R}_{CARDIAC}}\:and\:\:\frac{Q}{{P}_{MSF}-\:{P}_{RA}}\:=\:\frac{1}{{R}_{VR}}\:\:\:$$

$$\:solve\:both\:for\:Q;\:set\:equal\:to\:each\:other$$

$$\:\frac{{P}_{RA}}{{R}_{CARDIAC}}=\frac{{P}_{MSF}-\:{P}_{RA}}{{R}_{VR}}\:\:$$

$$\:solve\:for\:{P}_{RA}\:to\:obtain\:equation\:\left(1\right)$$

Preload unresponsive state:

$$\:\frac{Q}{{P}_{{RA}_{plat}}*}=\frac{1}{{R}_{CARDIAC}}\:and\:\:\frac{Q}{{P}_{MSF}-\:{P}_{RA}}\:=\:\frac{1}{{R}_{VR}}\:\:\:$$

$$\:solve\:both\:for\:Q;\:set\:equal\:to\:each\:other$$

$$\:\frac{{P}_{{RA}_{plat}}}{{R}_{CARDIAC}}=\frac{{P}_{MSF}-\:{P}_{RA}}{{R}_{VR}}\:\:$$

$$\:solve\:for\:{P}_{RA}\:to\:obtain\:equation\:\left(2\right)$$

$$\:{*P}_{{RA}_{plat}}\:is\:the\:{P}_{RA}\:at\:which\:the\:cardiac\:function\:curve\:flattens;$$

$$\:when\:the\:{P}_{RA}\:is\:above\:{P}_{{RA}_{plat}}\:the\:patient\:is\:preload\:unresponsive$$

## Abbreviations

PVPI: Portal vein pulsatility index
RV: Right ventricle
P_MSA_: Mean systemic pressure analogue
PR: Preload responsive
PUR: Preload unresponsive
T1–5: Time 1–5
RVEDA: Right ventricular end diastolic area
LVEDA: Left ventricular end diastolic area
RV S’: Tricuspid lateral annular systolic velocity
RVFAC: RV fractional area change
PAT: Pulmonary acceleration time
RIMP: Right myocardial performance index
LVOT VTI: Left ventricular outflow tract velocity time integral
LVEF: Left ventricular ejection fraction
LR: Lactated Ringer's solution
OP: Operating point
R_VR_: Resistance to venous return
R_CARDIAC_: Cardiac resistance or cardiac function
P_RA_: Right atrial pressure
Q: Blood flow - cardiac output or venous return
P_MSF_: Mean systemic filling pressure
P_PC_: Pericardial pressure
P_RA−plat_: Right atrial pressure when cardiac function curve forms plateau (i.e., flattens)
E_A_:E_ES_: Arterial elastance to end-systolic elastance
PLR: Passive leg raise
